# Evaluation of endogenous references for gene expression profiling in different tissues of the oriental fruit fly *Bactrocera dorsalis *(Diptera: Tephritidae)

**DOI:** 10.1186/1471-2199-11-76

**Published:** 2010-10-06

**Authors:** Guang-Mao Shen, Hong-Bo Jiang, Xiao-Na Wang, Jin-Jun Wang

**Affiliations:** 1 Key Laboratory of Entomology and Pest Control Engineering, College of Plant Protection, Southwest University, Chongqing 400715, P. R. China

## Abstract

**Background:**

Quantitative real-time reverse transcriptase PCR (RT-qPCR) has been widely used for quantification of mRNA as a way to determine key genes involved in different biological processes. For accurate gene quantification analysis, normalization of RT-qPCR data is absolutely essential. To date, normalization is most frequently achieved by the use of internal controls, often referred to as reference genes. However, several studies have shown that the reference genes used for the quantification of mRNA expression can be affected by the experimental set-up or cell type resulting in variation of the expression level of these key genes. Therefore, the evaluation of reference genes is critical for gene expression profiling, which is often neglected in gene expression studies of insects. For this purpose, ten candidate reference genes were investigated in three different tissues (midgut, Malpighian tubules, and fat body) of the oriental fruit fly, *Bactrocera dorsalis *(Hendel).

**Results:**

Two different programs, *geNorm *and *Normfinder*, were used to analyze the data. According to *geNorm*, α-TUB + ACT5 are the most appropriate reference genes for gene expression profiling across the three different tissues in the female flies, while ACT3 + α-TUB are considered as the best for males. Furthermore, we evaluated the stability of the candidate reference genes to determine the sexual differences in the same tissue. In the midgut and Malpighian tubules, ACT2 + α-TUB are the best choice for both males and females. However, α-TUB + ACT1 are the best pair for fat body. Meanwhile, the results calculated by *Normfinder *are quite the same as the results with *geNorm*; α-TUB is always one of the most stable genes in each sample validated by the two programs.

**Conclusions:**

In this study, we validated the suitable reference genes for gene expression profiling in different tissues of *B. dorsalis. *Moreover, appropriate reference genes were selected out for gene expression profiling of the same tissues taking the sexual differences into consideration. This work not only formed a solid basis for future gene expression study in *B. dorsalis*, but also will serve as a resource to screen reference genes for gene expression studies in any other insects.

## Background

Quantitative real-time reverse transcriptase PCR (RT-qPCR) has been widely used in gene expression analysis that provides insight into complex biological progresses [1]. This procedure of collecting data throughout the PCR process combines amplification and detection into a single step [2]. The advantages of this process include sensitivity, large dynamic range, and the potential for high throughout as well as accurate quantification [3].

Although RT-qPCR is often described as the gold standard, there are still some limitations of this assay such as reverse transcription and normalization [4,5]. A common technique in RT-qPCR is to normalize data by measuring the expression of a reference gene in the same samples in parallel. Housekeeping genes such as actin, tubulin, and glyceraldehyde-3-phosphate dehydrogenase (Gapdh) are usually used as an endogenous control for normalization to correct for amounts of starting material of RNA or differences in the cDNA synthesis efficiency. Although these genes have been defined functionally as "constitutively expressed to maintain cellular function," it does not necessarily meet the prerequisites for a good reference gene that can be "expressed at constant levels across all the experimental conditions, tissues or cell lines" [6-8]. Several studies have shown that some commonly used reference genes can be affected by the experimental set-up or cell type [7,9-13]. Each candidate reference gene should be evaluated under specific experimental conditions for gene expression profiling to make sure expression occurs at a constant level [14]. Furthermore, researchers have documented that multiple reference genes should be used for accurate normalization [15].

The oriental fruit fly, *Bactrocera dorsalis *(Hendel), is one of the most economically important fruit fly pests [16]. As a polyphagous species, this insect has the potential to invade new areas and to adapt to new host plants. The ramifications of the possible introduction in other economically significant fruit growing regions worldwide are cause for serious concern [17]. Molecular technology has already been widely used in previous studies of *B. dorsalis *[17-22], including some investigations of insecticide resistance [23-25]. As a major problem to limiting effective pest control, understanding resistance mechanisms at molecular levels is necessary. The midgut, Malpighian tubules, and the fat body are three major tissues found to play an important role in the metabolism and detoxification of xenobiotics in insects [26,27]. Several detoxifying enzymes involved in insecticide resistance, such as cytochrome P450s and glutathione-S-transferases, have been detected highly enriched in these insect tissues [26-29]. Exploring the gene expression profiles in these tissues will help our understanding of the resistance mechanisms [27]. The selection of suitable reference genes is a critical first step for the gene expression profiling in different tissues of *B. dorsalis.*

Several genes have been demonstrated to sex-differentially express in soma tissues of *Drosophila melanogaster *[30], and the genes related to insecticide resistance, such as P450, are regulated by female mating [31]. In addition, the number of female flies in the field is directly correlated with the degree of damage to the fruits; therefore, a comprehensive understanding of sexual differentiation may help the development of novel control mechanisms [19].

To date, few studies have been done to evaluate the stability of reference genes in entomological research [32,33]. Therefore, this study was undertaken to evaluate the ten candidate reference genes (ACT1, ACT2, ACT3, ACT5, 18S rRNA, GAPDH, G6PDH, α-TUB, β-TUB, and EF1α) in three different tissues (the midgut, Malpighian tubules, and the fat body) of *B. dorsalis *using RT-qPCR with SYBR Green using two different specific tools (*geNorm *and *Normfinder*), and thus provide appropriate reference genes to explore the gene expression patterns of the detoxifying and target enzymes in *B. dorsalis*.

## Results

All reference genes that can be downloaded from GenBank or have been cloned were considered as candidate reference genes. Therefore, in the present study, the expression stability of 10 reference genes (ACT1, ACT2, ACT3, ACT5, 18S rRNA, GAPDH, G6PDH, α-TUB, β-TUB, and EF1α) were evaluated in three different tissues (the midgut, Malpighian tubules, and the fat body) of both female and male adults of the oriental fruit fly (Table 1).

**Table 1:** Details of the primer pairs used for real-time PCR.

Gene name	GenBank accession number	Primer sequences (forward/reverse)	Amplicon length (bp)	Efficiency (%)	*R*^2^
GAPDH	GU269901	GACGCCTACAAGCCTGACAT GTTGAAGCGGGAATGATGTT	221	103.2	0.996
G6PDH	AB021910	CCTACAAACTTCTGCGGTTATGC AGAGCGAGGCGAGGTGATC	382	87.2	0.998
EF1α	GU269900	CGTTGGTGTCAACAAGATGG TGCCTTCAGCATTACCTTCC	230	107.6	0.957
18S	AF033944	GCGAGAGGTGAAATTCTTGG CGGGTAAGCGACTGAGAGAG	191	100.3	0.999
α-TUB	GU269902	CGCATTCATGGTTGATAACG GGGCACCAAGTTAGTCTGGA	184	104.8	0.994
β-TUB	EU938673	TTACATTTCTTTATGCCTGGTTTC CATTTGTTCGTCCACTTCCTTC	204	104.0	0.985
ACT1	L12253	AGCGTGAAATCGTGAGGGA GACAAGACCGAGTTGGCATA	286	98.2	0.999
ACT2	L12254	GTGTGATGGTTGGTATGGGA GGCTGGGGAGTTGAAGGTTT	269	89.5	0.998
ACT3	L12255	GGTCGGTATGGGACAGAAGG CTCACGATTGGCTTTTGGAT	220	94.5	0.996
ACT5	L12256	CAACTCACCCGCAATGTATG CGCTCAGCAGTGGTTGTAAA	237	100.6	0.996

*R*^2^, coefficient of determination.

### Total RNA quality

Total RNA was isolated from three different tissues of both the female and male adults. The dissociation curve had a single-peak and indicated a unique product of 382 bp by using the primers of G6PDH. This product crossed an intron of 609 bp on 1% agarose gel, indicating that the genomic DNA was completely removed from RNA samples. Spectrophotometric determination of total RNA concentration ranged from 113 to 1834 ng/μl. The A260:A280 value of the isolated total RNA ranged from 2.161 to 2.281, indicating the high purity of the total RNA. The 1% agarose gel electrophoresis was performed to confirm that the total RNA retained its integrity.

### Expression profiles of candidate reference genes

For each pair of primers, a dissociation curve with single-peak ensured that the primers amplified the unique product. The PCR efficiency and determination coefficient (*R*^2^) characterizing each standard curve is given in Table 1. The PCR efficiency of the 10 candidate reference genes was very good ranging from the lowest for G6PDH (87.2%) to the highest for EF1α (107.6%).

The raw *Cq *values ranged from 8.82 (18S) to 32.77 (ACT1) in the midgut; from 8.72 (18S) to 32.52 (ACT1) in the Malpighian tubules; and from 8.61 to 33.73 (ACT1) in the fat body. The smallest *Cq *variation in the midgut and in the Malpighian tubules was 18S rRNA with the value of 2.00 and 2.12, respectively; while in the fat body, ACT5 had a value of 1.57. ACT1 had the highest *Cq *variation both in the midgut and Malpighian tubules with the value of 4.37 and 6.70, respectively. The highest value in the fat body was 3.29 for β-TUB.

### Analysis of gene expression stability

#### *geNorm*

The *geNorm *program was applied to estimate the stability of the ten candidate reference genes among different tissues in both females and males, or in the same tissue between females and males. As the four actin genes belonged to the same functional class, they were analyzed separately with the other candidate genes. According to the *M *values calculated by *geNorm*, we ranked the candidate reference genes from the most to the least stable.

**Figure 1:**
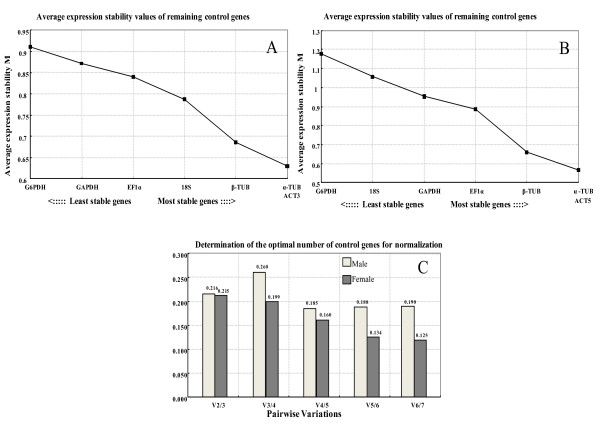
**Stability of candidate reference genes in different tissues of male (A) and female flies (B), and optimal number of reference genes for normalization (C) in *Bactrocera dorsalis *evaluated by *geNorm***. *geNorm *proceeds with stepwise exclusion of genes with relatively higher variable expression among the samples. The expression stability measure (*M*) is the average of the stability values of the remaining genes. The lower the *M *value, the more stable the gene in the subset. Bar values indicate the magnitude of the change in normalization factor after the inclusion of an additional reference gene. A large variation indicates that the added gene has a significant effect and should probably be included for calculation of the normalization factor. Same as in Figure 2.

The ranking of the candidate reference genes for male adults of oriental fruit files with their average *M *values from the lowest to highest was: ACT3 + α-TUB > β-TUB > 18S > EF1α > GAPDH > G6PDH, and the combination of ACT3 + α-TUB with the lowest *M *value (0.629) showed the greatest stability in males (Figure 1A).

However, the ranking of the candidate reference genes for female adults from the most stable to the least stable was: α-TUB + ACT5 >β-TUB >EF1α >GAPDH >18S >G6PDH (Figure 1B). With the lowest *M *value (0.564), the combination of α-TUB + ACT5 were considered to be the most stable reference genes when used in gene expression studies of different tissues. The lowest *M *values (0.564 and 0.629) for females and males were both lower than the default limit of *M *= 1.0 for heterogeneous tissues. The pairwise variation values were also calculated by *geNorm *and presented in Figure 1C.

**Figure 2:**
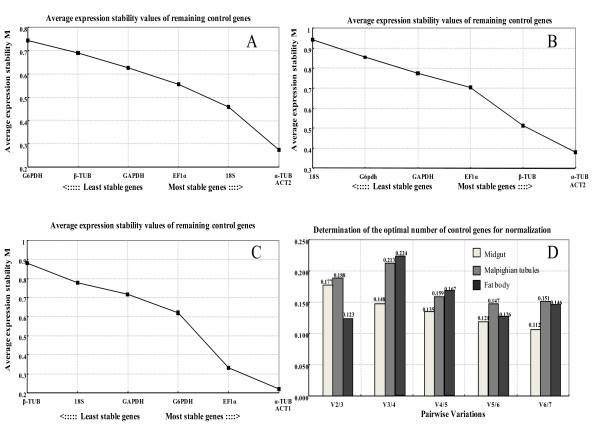
**Stability of candidate reference genes in sexual difference of midgut (A), Malpighian tubules (B), and fat body (C), and optimal number of reference genes for normalization (D) in *Bactrocera dorsalis *evaluated by *geNorm***.

In addition, we also determined if the reference genes were appropriate for determining sexual differences in the same tissue. The results showed that ACT2 + α-TUB with *M *= 0.274 displayed the most stability in the midgut according to *geNorm *analysis (Figure 2A). Similar to the midgut, ACT2 + α-TUB with *M *= 0.381 were also considered as the most stable pair in the Malpighian tubules (Figure 2B). In contrast, the evaluation of candidate reference genes for the fat body indicated that α-TUB + ACT1 with the lowest *M *value (0.219) were the most stable genes (Figure 2C). The lowest *M *value for each tissue was quite lower than the default limit value of 0.5 for homogeneous tissues. The pairwise variation values were also calculated by *geNorm *and presented in Figure 2D.

#### *Normfinder*

*Normfinder *was also used to investigate the suitable reference gene under experimental conditions. *Normfinder *ranked the various candidate reference genes according to their expression variation between inter- and intra-groups. The results showed that the ACT5 and ACT3 was the most stable gene among different tissues for female and male, respectively (Table 2). For gene selection that displays sexual difference in the same tissues, ACT5 was calculated to be the most stable in the midgut; in the Malpighian tubules, ACT3 was the most stable. The best choice for the fat body was EF1α (Table 3). Although the ranking was somewhat different from the results calculated by *geNorm*, α-TUB was considered one of the most stable genes in each sample calculated both by *geNorm *and *Normfinder*.

**Table 2:** Stability of candidate reference genes in different tissues of female and male *Bactrocera dorsalis *evaluated by *Normfinder*.

Ranking order	Gene name	Stability value of female	Gene name	Stability value of male
1	ACT5	0.246	ACT3	0.294
2	α-TUB	0.370	α-TUB	0.316
3	β-TUB	0.461	β-TUB	0.351
4	ACT3	0.477	18S	0.453
5	EF1α	0.522	EF1α	0.516
6	GAPDH	0.622	ACT5	0.543
7	ACT2	0.672	ACT1	0.571
8	18S	0.788	ACT2	0.584
9	ACT1	0.953	GAPDH	0.621
10	G6PDH	1.072	G6PDH	0.734

**Table 3:** Stability of candidate reference genes in sexual difference of the same tissues of *Bactrocera dorsalis *evaluated by *Normfinder*.

Ranking order	Gene name	Stability value in midgut	Gene name	Stability value in Malpighian tubules	Gene name	Stability value in fat body
1	ACT5	0.050	ACT3	0.180	EF1α	0.210
2	α-TUB	0.136	ACT2	0.200	ACT5	0.215
3	ACT2	0.170	α-TUB	0.240	ACT1	0.325
4	ACT3	0.277	EF1α	0.364	α-TUB	0.361
5	EF1α	0.295	β-TUB	0.463	G6PDH	0.398
6	18S	0.385	GAPDH	0.478	ACT2	0.440
7	GAPDH	0.431	ACT5	0.498	18S	0.494
8	β-TUB	0.458	ACT1	0.542	GAPDH	0.512
9	G6PDH	0.601	G6PDH	0.657	ACT3	0.641
10	ACT1	0.657	18S	0.816	β-TUB	0.755

## Discussion

As an accurate and sensitive method to detect the differentially expressed genes, RT-qPCR has contributed to understanding how developmental processes are conducted in a biological system [34]. When studying gene expression patterns in different tissues, a commonly used reference gene may be not stable under all experimental conditions [35]. The lack of really stable reference genes creates a greater risk of misinterpretation of results [36].

Recently, the selection of reliable reference genes has been taken into account in quantitative expression analysis. Such studies have been carried out in humans [37-40], animals [9,41-44], and plants [34,45-47]. However, this important aspect is often neglected in gene expression studies in insects [32,33,48]. Previous studies have been performed to find suitable reference genes that are stably expressed in different tissues of several species. The results indicated that an ideal reference gene among all different tissues may not exist [6,36,39,49,50].

In this study, multiple candidate reference genes were used to make the results more comprehensive. We searched GenBank and downloaded all the commonly used reference genes to obtain a comprehensive list of candidates. As there were only seven reference genes (ACT1, ACT2, ACT3, ACT5, G6PDH, β-TUB, and 18S) available, three more genes (α-TUB, EF1α, and GAPDH) were cloned to expand the number of the candidates. In total, ten genes were evaluated in this study by *geNorm *and *Normfinder*. When using *geNorm *to evaluate the stability of reference genes, genes belonging to the same functional class should not be analyzed together. Analyzing together may increase the chance that the genes are co-regulated [15]. Therefore, the four actin genes in this study were evaluated separately from six other genes. The rankings of *geNorm *and *Normfinder *were the same in female and male tissues. When evaluating the three tissues for sexual differentiation, although the results of two programs were not consistent with each other, α-TUB was always suggested as an optimal reference gene. Such discrepancies between programs are caused by using different mathematical models [51]. *Normfinder *takes all candidate genes into account and ranks the candidate genes with the minimal estimated intra- and inter-group variation. In contrast, *geNorm *sequentially excludes the worst gene, ending up with two genes and ranks genes with the degree of similarity of expression profile. The purpose of *geNorm *is to find the best two genes and provide information about the optimal number of genes in a given experimental condition. Unlike *Normfinder *considering all candidate reference genes for selection, the elimination processes of *geNorm *are based on the lower number of genes and the number decreases every cycle. *geNorm *is less sensitive to differentially expressed genes which can affect the results calculated by *Normfinder. *These factors may lead to differences in calculation results. Our findings also suggested that among different tissues or within the same tissues between females and males, the candidate reference genes are actually variable in expression. Commonly used reference genes may be not as stable as they were thought under certain experimental condition, thus the evaluation of reference genes is necessary. According to the calculation using *geNorm*, the most suitable reference genes within different female tissues are α-TUB + ACT5. However, for male tissues, α-TUB + ACT3 are the most stable reference genes. Furthermore, we found that the rankings of candidate reference genes are quite different. This may suggest that sexual differences should be considered when evaluating reference stability. Consequently, the differences between female and male could be an important aspect for future study to improve our understanding of this species.

Since we did find stability differences between females and males, we also evaluated the stability of candidate reference genes in the same tissue between females and males. The most stable pairs of reference genes calculated by *geNorm *in the midgut, Malpighian tubules, and the fat body were ACT2 + α-TUB, ACT2 + α-TUB, and α-TUB + ACT1, respectively. Here we see the *M *and V_n_/V_n+1 _values in the same tissues are quite lower than in different tissues, which indicates that the reference genes are much more stable in homogeneous tissues than in heterogeneous tissues. In fact, previous studies have recommended that when using *geNorm *to estimate reference genes in different tissues, the acceptable *M *values for homogeneous tissues should be less than 0.5 and for heterogeneous tissues and cancer samples should be less than 1.0 [52].

Although 18S rRNA was highly expressed in all samples with the lowest *Cq *values ranging from 8.61 to 10.83, its *M *value is one of the largest either in females or males. This indicates that 18S rRNA is not suitable as a reference gene under our experimental conditions. This result is in line with the earlier studies that 18S rRNA is not stable enough under specified experimental conditions [9,32,36,43,53]. The transcription by a separate RNA polymerase was thought to be a reason why rRNA could not be considered as a stable reference gene [54]. However, other studies concluded that 18S rRNA was suitable for tissue analysis or could be combined with other genes as reference genes [55,56]. Our results strongly suggest that when18S rRNA is used as a reference gene, validation of its stability must be carried out to avoid errors caused by normalization.

Even for housekeeping genes, whose products are indispensable for every living cell and which are relatively stably expressed, there are tissue-specific differences based upon extra demands in the required rate at which new housekeeping proteins need to be produced to maintain cell function [7]. In our study, α-tubulin and β-tubulin were chosen as candidate reference genes. According to the calculation by both *geNorm *and *Normfinder*, α-tubulin is much more stable than β-tubulin across all tissues, especially in the fat body.

Since there are still only a limited number of reference genes of *B. dorsalis *that can be used in evaluation, more reference genes need to be cloned to use as candidates. Meanwhile, increasing the number of samples will make the results more accurate. To enable thorough evaluation and efficient repeatability of our results, we have added the complete information followed by the Quantitative Real-Time PCR Experiments (MIQE) guidelines, which is set up to ensure the integrity of the scientific literature, promote consistency between laboratories, and increase experimental transparency [57].

## Conclusions

In conclusion, we demonstrated that not all reference genes are stably expressed across different tissues and between sexes of insects. We verified the caution that housekeeping genes should be evaluated for gene expression profiling under specified experimental conditions when used as a reference gene. In the current study, we validated the possible suitable reference genes for gene expression profiling in different tissues (the midgut, Malpighian tubules, and the fat body) of *B. dorsalis*. Moreover, appropriate reference genes were selected for gene expression profiling of the same tissues (the midgut, Malpighian tubules, and the fat body) taking the sexual differences into consideration. Our work has formed a solid basis for future study on the expression profiles of insecticide resistance related genes of *B. dorsalis *at molecular levels.

## Methods

### Insects

The oriental fruit fly, *Bactrocera dorsalis*, was originally collected from Yunnan province, People's Republic of China. The adults were reared in glass cages and fed on an artificial diet consisting of yeast powder, honey, sugar, vitamin C, and water. Every two days, a banana was put into the cage to collect eggs. After hatching, larvae were reared on banana in plastic basins with sand until pupation. Whole life stages were kept in a temperature controlled room at 27 ± 1°C, 70 ± 5% relative humidity and photoperiod cycle of 14 h L/10 h D.

### Collection of different tissues

The flies within 10 days after eclosion in the same season were used. Ten adult males and ten adult females were dissected individually using dissection needle in physiological saline under a stereomicroscope (Olympus SZX12, Japan). The midgut, Malpighian tubules, and the fat body were collected separately and placed in a 1.5 ml centrifuge tube. Subsequently, the collected tissues were used for RNA extraction after a quick freezing in liquid nitrogen. Four replicates were run for the female group and three replicates for the male group.

### Isolation of tissue RNA and synthesis of cDNA

Collected tissues were homogenized immediately after dissection with liquid nitrogen in a mortar. RNA was extracted following the manufacturer's instruction for the RNeasy plus Micro Kit (QIAGEN). RNA was quantified by measuring the absorbance at 260 nm using a NanoVue UV-Vis spectrophotometer (GE Healthcare). The purity of all RNA samples was assessed at an absorbance ratio of OD_260/280 _and OD_260/230_, and the integrity of RNA was checked with 1% agarose gel electrophoresis. Total RNA extraction also included a genomic DNA elimination step by using a genomic DNA elimination column, which could efficiently remove genomic DNA. To insure that there was no genomic DNA contamination, the G6PDH primers, which crossed a 609 bp intron, was used to amplify the synthesized cDNA in RT-qPCR reaction.

The first strand cDNA was synthesized from 500 ng of DNA-free RNA isolated from either the midgut, Malpighian tubules or the fat body using the PrimerScript™ RT Reagent Kit Perfect Real time Kit (Takara) according to the manufacturer's instructions. Briefly, the 10 μl reaction system was composed of 500 ng RNA, 200 pmol Random 6 mers, 2 μl reverse transcription buffer, 0.5 μl PrimerScript™ RT Enzyme Mix I and RNase free dH_2_O. The reverse transcription reaction was performed using a TGradient PCR Thermal Cycler (Biometra). The reaction condition included a step of 37°C for 15 min and 85°C for 5 sec. After the reverse transcription, synthesized cDNA was stored at -20°C for future use.

### Quantitative Real-time PCR

Primer 3 software (version 0.4.0) [http://frodo.wi.mit.edu/primer3/] and Primer 5.0 [http://www.premierbiosoft.com/] were used to design primers. According to the results of two programs, two pairs of the primers were selected and used for the reaction; the pair with better efficiency and single PCR product was then used in the experiments. All primers were placed in continuous exons except the G6PDH pair, which was separated by a 609 bp intron. For RT-qPCR, ten reference genes total were evaluated for the midgut, Malpighian tubules, and the fat body. Primers, amplicon sizes, optimal annealing temperature, and PCR efficiencies and coefficients of determination are presented in Table 1.

All reactions were performed on a Stratagene Mx3000P thermal cycler (Stratagene). The 25 μl reaction system consisted of 2 μl of diluted cDNA, 12.5 μl SYBR^® ^Green Realtime PCR Master Mix (QPK-201) (Toyobo) and 0.2 mM of each primer. Thermal cycling conditions were: an initial denaturation at 94°C for 1 min, followed by 40 cycles of 94°C for 15 sec, 60°C for 30 sec, and 72°C for 30 sec. After reaction, a melting curve analysis from 55°C to 95°C was applied to all reactions to ensure consistency and specificity of the amplified product. In addition, the amplification size was checked by running an agarose gel electrophoresis of the PCR product for each primer pair. A 10-fold dilution series of cDNA from midgut was employed as a standard to construct a relative standard curve and determine the PCR efficiency that would be used in converting quantification cycle (*Cq*-values) into raw data (relative quantities).

### Data analysis

The amplification efficiency of reaction was calculated by the MxPro 4.01 software for Mx3000P (Stratagene) based on dilution curves.

The *geNorm *program [http://medgen.ugent.be/~jvdesomp/genorm/] was used to calculate the mean pair-wise variation between an individual gene and all other tested candidate reference genes and the results were shown as expression stability (*M*). Candidate genes with the lowest *M *value were considered to be most stably expressed under tested experimental conditions, and thus could be selected as ideal reference genes. In calculation, at each step, the least stable reference gene was removed and the average expression stability value of remaining reference genes was calculated until the two most stable genes were left (which cannot be further calculated). Furthermore, a value of V_n_/V_n+1 _was also given to show the pair-wise variation between two sequential normalization factors containing an increasing number of genes. Large variation indicated that the added gene has a significant effect and should be included for calculation of a reliable normalization factor [15].

"*Normfinder*" [http://www.mdl.dk/publicationsnormfinder.htm] is an algorithm for identifying the optimal normalization gene among a set of candidates. The strategy is rooted in a mathematical model of gene expression that enables estimation not only of the overall variation of the candidate normalization genes but also of the variation between samples subgroups of the sample set [51].

Raw *Cq *values were converted to relative quantities using the comparative *Cq *method with a procedure of specific PCR efficiency correction and then transformed to an input file format suitable for subsequent analysis by the *geNorm *and *Normfinder *Excel applications.

## List of Abbreviations

RT-qPCR: quantitative real-time reverse transcriptase PCR; GAPDH: glyceraldehyde-3-phosphate dehydrogenase; G6PDH: glucose-6-phosphate dehydrogenase; EF1α: elongation factor-1 alpha; 18S: 18S rRNA; α-TUB: alpha tubulin; β-TUB: beta 2-tubulin; ACT1: muscle-specific actin (clones pBdA1 and pBdA1.2); ACT2: muscle-specific actin (clones pBdA2.1 and pBdA2); ACT3: muscle-specific actin (clone pBdA3); ACT5: muscle-specific actin (clone pBdA5).

## Authors' contributions

GMS carried out the RT-qPCR experiments, participated in data analysis and drafted the manuscript. HBJ analyzed the data, participated in the design of the study and drafting of the manuscript. XNW helped to execute the RT-qPCR experiments and participated in data analysis. JJW supervised the process, contributed to the design of the study and drafting of the manuscript. All authors read and approved the final manuscript.
